# A Simulation Framework for Zoom-Aided Coverage Path Planning with UAV-Mounted PTZ Cameras

**DOI:** 10.3390/s25175220

**Published:** 2025-08-22

**Authors:** Natalia Chacon Rios, Sabyasachi Mondal, Antonios Tsourdos

**Affiliations:** Faculty of Engineering and Applied Science, Cranfield University, College Rd., Wharley End, Bedford MK43 0AL, UK; nrcr.28@hotmail.com (N.C.R.); a.tsourdos@cranfield.ac.uk (A.T.)

**Keywords:** coverage path planning, unmanned aerial vehicles, pan–tilt–zoom cameras, ground sampling distance, energy efficiency, zoom compensation, terrain-aware imaging, sensor-based adaptation

## Abstract

Achieving energy-efficient aerial coverage remains a significant challenge for UAV-based missions, especially over hilly terrain where consistent ground resolution is needed. Traditional solutions use changes in altitude to compensate for elevation changes, which requires a significant amount of energy. This paper presents a new way to plan coverage paths (CPP) that uses real-time zoom control of a pan–tilt–zoom (PTZ) camera to keep the ground sampling distance (GSD)—the distance between two consecutive pixel centers projected onto the ground—constant without changing the UAV’s altitude. The proposed algorithm changes the camera’s focal length based on the height of the terrain. It only changes the altitude when the zoom limits are reached. Simulation results on a variety of terrain profiles show that the zoom-based CPP substantially reduces flight duration and path length compared to traditional altitude-based strategies. The framework can also be used with low-cost camera systems with limited zoom capability, thereby improving operational feasibility. These findings establish a basis for further development and field validation in upcoming research phases.

## 1. Introduction

### 1.1. Motivation

Energy efficiency remains a fundamental limitation in deploying unmanned aerial vehicles (UAVs) for large-scale or time-sensitive missions. In coverage tasks such as agricultural mapping, environmental surveillance, and disaster assessment, UAVs must balance high-resolution data acquisition with battery constraints and terrain irregularities. Traditional solutions adapt the UAV’s altitude to preserve image resolution when flying over undulating terrain; however, this results in frequent vertical motion, which increases energy consumption and mechanical wear.

Camera systems equipped with pan–tilt–zoom (PTZ) capabilities offer an untapped opportunity—PTZ systems allow dynamic adjustment of the focal length (zoom) to maintain a desired ground sampling distance (GSD) without requiring altitude variation. This shift from UAV-based to sensor-based adaptation can reduce energy consumption, create smoother flight paths, and enable longer missions. Despite its potential, the research community has given almost no attention to zoom integration in coverage path planning (CPP), highlighting a clear opportunity for innovation.

### 1.2. Literature Review

In recent years, unmanned aerial vehicles (UAVs) have been increasingly employed across a range of applications, like infrastructure monitoring, search and rescue missions (SAR), and environmental protection. These applications require solving coverage path planning (CPP) problems, which focus on identifying optimal trajectories for the UAV to take so that it can cover the whole area while keeping in mind how long it can fly, how much energy it has, and how well it can see. Numerous researchers have worked on CPP, which takes energy into account. Fevgas et al. [1] and others discussed how important it is to save energy and how easy it is to sweep flat ground with basic sweep patterns. Their study highlights the criticality of finding the right balance between resolution, full coverage, overlap, and energy usage. Previous CPP works thought that the camera settings and the height of the flights would always be the same, leading to a different ground sample distance (GSD) for distinct types of ground. This difference may hinder post-processing and interpretation, and the analysis that comes after them may be less helpful. Researchers have been looking for a way to solve the problem by combining adaptive systems that change the camera settings in real time to keep the GSD steady. One way to keep the GSD steady is to change the UAV’s height in real time to match the changes in the ground. But this method might use more energy and make it harder to fly, especially in places where the altitude changes severely [1,2]. Recent CPP approaches have also integrated real-time terrain mapping using LiDAR or SLAM to dynamically adapt altitude or sensor parameters, even in GPS-denied or unknown environments [3,4]. Energy savings can also be achieved using pan–tilt–zoom (PTZ) cameras due to their variable focal length *f*. UAVs can adjust the focus length to keep a steady GSD when the ground is wavy. Hence, they do not need to change the altitude.

Kazemdehbashi and Liu [5] proposed a CPP algorithm to enhance SAR effectiveness for UAVs flying in windy conditions. In the same way, Alpdemir and Sezgin [6] proposed a hybrid approach that combines RL with a ground-penetrating radar for UAV path planning to facilitate terrain visualisation and find buried objects. Mu et al. [7] came up with an improved back and forth (IBF) coverage mode that makes the path shorter, the calculations faster, and the movement easier. Their method mitigated limitations associated with traditional CPP methods like cellular decomposition (CD) and probabilistic roadmap (PRM). Heydari et al. [8] also used RL to solve the NP-hard (non-deterministic polynomial-time hardness) CPP problem for unknown environments. They came up with a hybrid RL strategy that combines basic zigzag paths with an RL agent. This improves coverage and reduces computational demand.

Despite the significant benefits, the integration of focal length adaptation in real time into UAV systems remains an underexplored area. Most implementations rely on static focal length settings or discrete zoom levels, which are often inadequate for dynamic environments [9]. Nevertheless, recent studies have begun to bridge this gap. For example, Wu et al. [10] proposed an optical system design that maintains constant ground resolution by adjusting the focal length in response to varying viewing geometries. Their approach uses freeform optical surfaces to enhance aberration control and achieve consistent GSD across diverse terrains.

In parallel, other studies focus on real-time adaptive gimbal and zoom control to optimise ground resolution in dynamically changing topographies [11].

### 1.3. Main Contributions

This paper addresses the aforementioned gap by presenting a zoom-aware CPP framework that unifies path planning and real-time focal length adjustment. The main contributions of this work are as follows:

- Zoom-integrated CPP algorithm: A novel method that dynamically adjusts the focal length according to the terrain to maintain constant ground resolution without altitude changes.
- Energy-efficient path planning: Demonstration that zoom-based compensation significantly reduces path length and flight time compared to altitude-only approaches.
- Hybrid strategy for constrained zoom systems: An adaptable model that integrates altitude changes only when zoom limits are exceeded, enabling the use of lower-end PTZ cameras.
- Comprehensive evaluation: Simulations on synthetic terrains of varying complexity demonstrate robustness, resolution consistency, and operational gains over existing CPP strategies.

## 2. Materials and Methods

### 2.1. Boustrophedon Path: Optimised Sweep Path for Camera Coverage

The Boustrophedon method creates a path that is a structured area coverage strategy. This method is often used in aerial surveys and robotic mapping. This method has the vehicle moving back and forth through a region of interest (ROI), resembling a ploughing pattern used in agriculture. The term Boustrophedon originates from an ancient script style in which lines of text are written alternately from left to right and right to left [12]. Within the framework of coverage path planning (CPP) [12], the Boustrophedon path comprises two principal components:

- Straight sweep segments: These linear trajectories represent the portions of the path during which the vehicle acquires imagery. The primary objective during these segments is to achieve comprehensive coverage with a predefined image overlap [12].
- Turning manoeuvres: These manoeuvres occur at the transition points between consecutive sweep lines. The design and execution of these turns have a direct impact on overall mission duration and energy consumption [12].

The lateral spacing between successive sweep lines constitutes a key parameter in defining the path’s ability to effectively cover the ROI. This spacing is derived based on the specifications of the onboard camera system and the desired image overlap. In particular [12]:

- Camera specifications: The horizontal field of view (FOV) and pixel resolution determine the ground width captured in each image.
- Ground sample distance (GSD): Defined as the ground distance represented by a single image pixel (typically in meters per pixel), the GSD is influenced by both sensor resolution and flight altitude.
- Image side lap: This parameter defines the percentage of overlap between adjacent images along the sweep axis. Sufficient side lap is essential to ensure continuous coverage and to support subsequent image processing or stitching.

To facilitate a tractable analysis of flight duration and image acquisition, the following simplifying assumptions are adopted:

- Ideal path adherence: It is presumed that the vehicle follows the planned trajectory precisely, thereby streamlining the modelling process.
- Uniform airspeed: The vehicle is assumed to maintain a constant velocity throughout the mission, typically governed by an autopilot system.

This method of Boustrophedon path planning offers a time-efficient and methodical approach to area coverage. By carefully tuning parameters such as sweep spacing and survey orientation, it becomes possible to ensure complete ROI coverage with the required image overlap. The assumptions made contribute to the practical feasibility of mission planning, rendering this approach particularly suitable for aerial survey applications under known environmental conditions [12].

### 2.2. Boustrophedon Path: Optimised Sweep Path for Camera Coverage with Zoom Implementation

In this section, an optimised CPP algorithm basis is presented to ensure complete terrain coverage while maintaining a constant ground resolution by dynamically adjusting the camera’s *f*. The approach begins by defining camera intrinsic parameters, such as sensor dimensions and the allowable range *f*, alongside the desired ground resolution. The image footprint is derived from these values, which directly inform the UAV’s movement increments in both the horizontal (*X*) and vertical (*Y*) directions. Image overlap between adjacent shots is also incorporated to ensure seamless coverage without any gaps.

A synthetic terrain with sinusoidal elevation is used to test the zoom-based coverage algorithm. At each waypoint, the UAV calculates the required focal length to maintain the target resolution. Focal length is computed from standard field-of-view geometry using the UAV-to-ground distance and desired resolution. If the result exceeds the camera’s zoom limits, the UAV adjusts its altitude. This adaptive mechanism ensures resolution consistency while minimising altitude changes (see Algorithm 1).

- To achieve the desired ground resolution, the system first attempts to adjust the camera *f* within its mechanical limits.
- Focal length *f* adjustment and limits: The camera zoom system works by adjusting the (*f*), which is constrained by the mechanical limits of the camera:
  (*f*) must stay within the range 0.0043 m < *f* < 0.129 m.
  If *f* remains within this range, the UAV maintains its current altitude and achieves the target resolution only through zoom adjustments.
  If *f* falls outside these limits, altitude adjustment becomes necessary.
- If the camera cannot achieve the required resolution solely by changing its *f*, the UAV must adjust its altitude according to the GSD, which is the real-world distance represented by a single pixel in the captured image.
  The required UAV altitude (H) is derived using the field-of-view equation (as shown in Line 4 of Algorithm 1), plus the altitude of the terrain. This ensures that the UAV adjusts its altitude only when zoom limits are exceeded.

where $R_{v}$—the vertical resolution of the camera in pixels, $r_{d}$—the desired ground resolution (m/pixel), $w_{s}$—the camera sensor width (m), $f_{\min},f_{\max}$—the minimum and maximum focal lengths (m), $h_{\mathrm{terrain}}$—the terrain elevation (m), $h_{\mathrm{base}}$—the UAV base altitude (m), and *f*—the calculated focal length (m). To ensure full coverage, the UAV follows a boustrophedon zigzag pattern. The UAV proceeds along the X-axis. When it reaches the edge of the terrain, it executes a turning manoeuvre and advances to the next *Y*-row. Boundary conditions are set up so that movement does not go beyond the physical limits of the terrain. For example, if the next calculated *X* position goes beyond the edge of the terrain, the UAV stays at the current *X* position and adds to the *Y* coordinate. This makes sure that the whole grid is scanned in a way that makes sense.

Algorithm 1 performs real-time focal length adjustment to maintain consistent GSD across varying terrain elevations. The algorithm executes lightweight trigonometric calculations based on terrain altitude data, which are computationally efficient. Each iteration performs a fixed number of operations, like altitude lookup, focal-length calculation, and conditional logic.
**Algorithm 1** Dynamic altitude adjustment for valid focal length.1:**Input:** $R_{v}$, $r_{d}$, $w_{s}$, $f_{\min}$, $f_{\max}$, $h_{\mathrm{terrain}}$
2:$Z\leftarrow h_{\mathrm{base}}$▹ Initial UAV altitude3:$d\leftarrow Z-h_{\mathrm{terrain}}$▹ Distance to ground4:$\mathrm{FOV}\leftarrow 2\;\cdot \;\arctan\left(\frac{r_{d}\;\cdot \;R_{v}}{2\;\cdot \;d}\right)$
5:$f\leftarrow \frac{w_{s}}{2\;\cdot \;\tan(\mathrm{FOV}/2)}$
6:**if** $f_{\min}\leq f\leq f_{\max}$ **then**
7:    **return** *Z*▹ Focal length is within limits8:**else**
9:    **if** $f<f_{\min}$ **then**
10:        $\mathrm{FOV}\leftarrow 2\;\cdot \;\arctan\left(\frac{w_{s}}{2\;\cdot \;f_{\min}}\right)$
11:    **else**
12:        $\mathrm{FOV}\leftarrow 2\;\cdot \;\arctan\left(\frac{w_{s}}{2\;\cdot \;f_{\max}}\right)$
13:    $d\leftarrow \frac{r_{d}\;\cdot \;R_{v}}{2\;\cdot \;\tan(\mathrm{FOV}/2)}$
14:    $Z\leftarrow h_{\mathrm{terrain}}+d$
15:    $f\leftarrow \frac{w_{s}}{2\;\cdot \;\tan(\mathrm{FOV}/2)}$
16:    **return** *Z*


Regarding latency, the focal length adjustment is intended to run onboard during flight at camera frame intervals. Based on MATLAB R2024a profiling, the focal length computation and altitude adjustment take less than 1 ms per waypoint on a standard laptop. When ported to embedded flight controllers as ArduPilot or PX4, the latency introduced by zoom commands via PWM or MAVLink is in the range of tens of milliseconds [10], which is acceptable within the 1-s photo interval used in the simulation [13].

The flight control feedback loop is assumed to operate independently through the UAV’s autopilot, which maintains fixed altitude and heading between waypoints. Zoom adjustments do not interfere with the autopilot loop but are instead issued as periodic commands to the gimbal system. In real-world integration, these commands would be triggered through onboard mission scripts [13]. When zoom limits are exceeded, the algorithm triggers altitude changes, which are then reflected in the flight controller’s guidance loop—this behaviour is simulated using terrain-aware updates in the z-axis.

In the simulation framework, the UAV estimates its real-time altitude above the terrain using a terrain elevation grid, which serves as a synthetic digital elevation model [14]. At each waypoint in the coverage path, the UAV retrieves the underlying terrain elevation by rounding its current x-y position to the closest grid coordinates. The vertical distance between the UAV and the terrain is then computed as the difference between the UAV’s altitude and the terrain elevation at that point. This inferred distance is used to calculate the required field of view and focal length to achieve the target ground sampling distance (GSD). Although this approach assumes perfect knowledge of terrain geometry, it reflects the kind of altitude awareness that can be achieved in real-world operations using onboard sensors like LiDAR, radar altimeters, stereo cameras, or a fused DEM-GNSS solution [15].

The UAV also uses trigonometric relationships to figure out the yaw, pitch, and roll angles at each step based on the spatial separation between consecutive waypoints. The UAV’s angular velocities are calculated by looking at how quickly these angles change over time. This facilitates verification of the UAV’s rotational dynamics and ensures that transitions are smooth, especially when it turns or changes altitude. Throughout the simulation, cumulative metrics such as total distance travelled, total flight time, and number of directional turns are computed to evaluate the performance and efficiency of the coverage strategy.

### 2.3. UAV Velocity Model

To ensure the UAV captures images with the correct amount of overlap, it is necessary to determine the UAV’s speed while minimising redundancy. For tasks like photogrammetry, where overlapping images are needed to make accurate 3D models or mosaics of the terrain, this overlap is very important [16]. Setting the overlap to 0% in the CPP ensures a comprehensive and logical scanning of the entire grid. This is important in search operations where the entire area should be scanned as quickly as possible [1].

For an accurate photogrammetric reconstruction, it is important that the overlap between successive aerial images is always the same. To do this, the UAV’s forward speed needs to be controlled based on how often it takes pictures and how far it travels on the ground between shots. This link allows the UAV to keep the target overlap percentage, which is important for obtaining good survey results. Using the following equation [17], the required speed can be calculated using the following:

(1) $$S_{o}=\frac{F_{vi}\left(100-O_{v}\right)}{100(I_{p}+0.1)}$$

where $S_{o}$ is the speed at a desired image overlap, $F_{vi}$ is the vertical image footprint distance, $O_{v}$ is the desired vertical image overlap, and $I_{p}$ is the photo shooting interval. For this study, a constant UAV speed of 3.4727 m per second is adopted to enable comparison with the previous work presented in [18].

### 2.4. Simulation Data

#### 2.4.1. Camera Data

To choose a PTZ camera that would work well with UAVs, this study examined several options and compared them based on size, weight and sensor resolution. The Drone Pan Tilt Gimbal Camera U30T was chosen because it had technical documentation, was easy to integrate, and met the project’s goals.

While other cameras offered additional features such as thermal imaging, these were not required for this implementation. However, the developed algorithms are designed to be modular and can be adapted to alternative camera modules by modifying the relevant input parameters in the system. The PTZ camera specifications used in this study are presented in Table 1, providing key sensor and optical characteristics used in simulation calculations [19].

#### 2.4.2. PTZ Integration and Zoom Control

In a practical deployment scenario, the PTZ camera is mounted on a stabilised gimbal attached to the UAV’s payload bay or underside frame. The camera used in this study is designed for UAV integration and supports standard interfaces for command and control, including serial (UART), IP, or PWM protocols. The dynamic adjustment of focal length (zoom) is achieved by sending control signals from the onboard flight controller to the PTZ unit. These signals specify the required zoom level based on real-time altitude and resolution computations. Although the current study evaluates the zoom strategy in simulation, the selected PTZ camera model is fully compatible with popular flight stacks such as ArduPilot and PX4, which allows direct zoom control of the camera in real time during flight operations.

## 3. Results and Discussion

The methodology described above establishes a robust framework for achieving consistent ground resolution using a combination of camera zoom adjustments and dynamic altitude control. By prioritising focal length adaptation within the mechanical limits of the imaging system and employing altitude changes only when necessary, the proposed approach optimises both coverage quality and energy efficiency. A series of simulations was run on synthetic terrain profiles of different levels of difficulty to evaluate the effectiveness of this hybrid strategy in real life. The next part shows and talks about the results of these tests. It compares the baseline altitude-only method with the suggested focal-length-aware method in terms of how well it works for paths, how well it keeps the resolution of the images, and how well the UAV can move around.

### 3.1. Boustrophedon CPP: Comparison Between Altitude Adjustment and Zoom Compensation for Uniform Camera Coverage

The following two approaches were evaluated to cover the Boustrophedon CPP to ensure that the ground resolution stayed at 50 pixels per metre: (1) altitude adjustment and (2) zoom compensation. These methods were tested on three different synthetic terrain profiles with different elevation features to assess image quality retention and flight efficiency.

**Figure 1:**
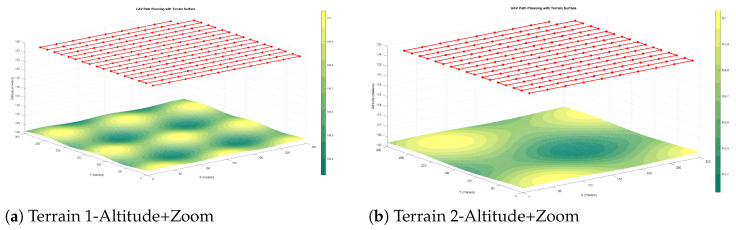
Proposed zoom-based compensation strategies over Terrain Types 1 and 2. The zoom-based method achieves uniform ground resolution with reduced altitude variation and energy use.

In the altitude adjustment approach, the UAV dynamically varies its flight altitude in response to terrain elevation, compensating for height variations to preserve uniform resolution. Figure 1a,b illustrate the resulting paths over

- Terrain Type 1—smooth elevation profile;
- Terrain Type 2—moderate undulations.

The results confirm the method’s ability to maintain constant resolution by adapting flight height. However, the resulting trajectories tend to increase energy consumption and flight time due to frequent altitude changes.

The zoom compensation method minimises altitude changes by adjusting the focal length dynamically. As shown in Figure 1, this results in smoother trajectories and reduced energy consumption compared to altitude-only planning. (See Figure 13 in [18]).

**Figure 2:**
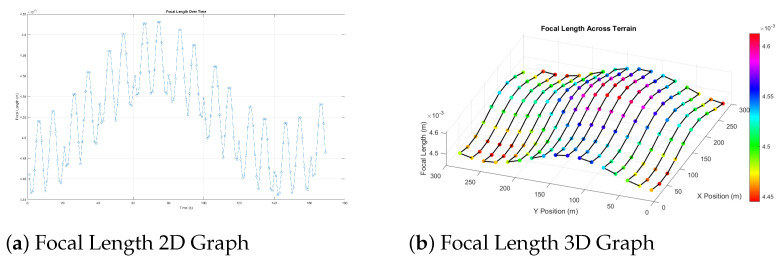
(**a**) Two dimensional (2D) and (**b**) 3D visualisations of how the focal length (zoom) changes at each waypoint during flight over Terrain Type 2. The UAV maintains a fixed altitude while adjusting zoom to preserve GSD.

Figure 2 shows how the focal length changes along the CPP path to illustrate the zoom adjustment mechanism. These changes are related to changes in the terrain and show that the system can respond in real time. Both methods work well to obtain the same ground resolution overall. The zoom-based solution is more practical for deployment, offering improved efficiency without requiring frequent altitude changes.

### 3.2. Performance Evaluation of Zoom-Based vs. Altitude-Based CPP

Table 2 presents a quantitative comparison of two CPP strategies applied to Terrain Type 2, the traditional altitude-based approach and the newly proposed zoom-based approach. Both methods use the same number of turns (16) and waypoints (170), ensuring a fair comparison under identical coverage constraints.

The zoom-based CPP shows clear advantages. Although the total flight distance is only slightly reduced (from 4976.66 to 4945.92 m), the total flight time decreases significantly, from 228.09 s to 169.00 s, representing an approximate 26% reduction in mission duration. This time saving is primarily due to the constant-altitude flight enabled by the zoom adaptation, which avoids energy-intensive altitude changes and results in smoother, more efficient navigation.

These results validate the effectiveness of integrating zoom control in CPP for UAV-based search operations. Compared with prior implementations that relied solely on altitude modulation, the zoom-enhanced method not only conserves flight time and potentially energy but also simplifies flight dynamics by maintaining a consistent altitude throughout the mission.

Although the proposed framework is intended primarily for real-time monitoring and energy-efficient aerial coverage, the algorithm maintains a consistent ground sampling distance (GSD). However, variations in focal length can introduce differences in image scale and optical distortion, which can introduce difficulties in image scale or perspective, affecting applications like stitching or 3D reconstruction. These challenges can be addressed by recording per-image focal-length metadata and applying correction techniques during post-processing.

### 3.3. Extended Terrain Analysis: Mixed Elevation Terrain with Zoom-Based CPP

**Figure 3:**
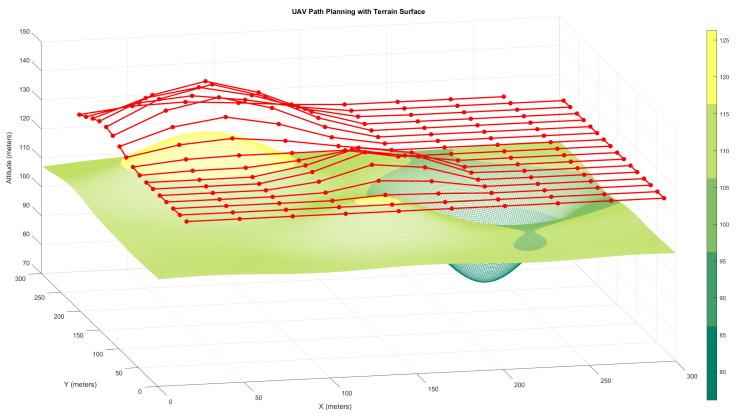
Simulated UAV path using the zoom-based coverage path planning method over terrain with mixed elevation features. The UAV maintains a constant altitude while dynamically adjusting focal length to preserve image resolution.

To test the zoom-based CPP method even more, another simulation was run using a terrain profile with both sharp peaks and deep valleys. This complex surface was used to assess the UAV’s ability to keep the same level of detail over terrain that changes a lot while keeping the altitude changes to a minimum. Figure 3 shows the generated UAV path over this terrain using the zoom-compensated approach. Despite dramatic elevation changes, the UAV successfully maintains a fixed flight altitude and dynamically adjusts its *f* to maintain a constant ground resolution of 50 pixels per meter.

**Figure 4:**
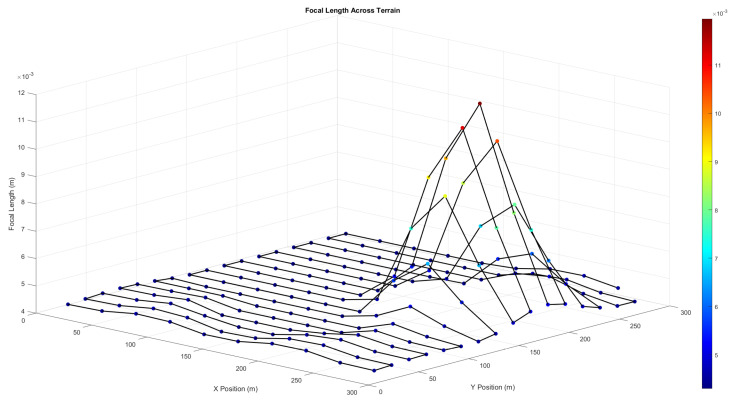
Required focal length values at each waypoint across complex terrain. Higher focal lengths are observed over elevated regions, illustrating adaptive zoom behaviour to ensure uniform imaging resolution.

To better understand the zoom behaviour over varied terrain, Figure 4 shows the *f* values required at each waypoint to maintain a consistent resolution of 50 pixels per metre. Sharp terrain changes result in higher *f* demands, peaking over elevated areas while reducing over valleys.

The UAV must adjust its imaging parameters within hardware limits to ensure this resolution throughout the mission. Given the sensor and camera specifications (*f* limits and sensor width (Section 2.4.1), the corresponding operational altitude range is shown in Table 3.

These limits set the vertical envelope that the UAV must stay within. The zoom-based CPP can move around this range without changing its altitude, which keeps the resolution high and the energy use low. The total distance travelled in this case is 5014.95 m, and the total flight time is 169.00 s. These results are similar to the Terrain Type 2 previously analysed, which looked at the number of turns and the time it took to fly. This shows how adaptable the CPP method is when using Zoom. The UAV flies smoothly and efficiently even when the terrain varies and keeps its altitude. These findings suggest the method is suitable for real-world deployment in challenging environments where precision and energy saving are needed.

### 3.4. Evaluating Model Performance with Reduced Maximum Focal Length

**Figure 5:**
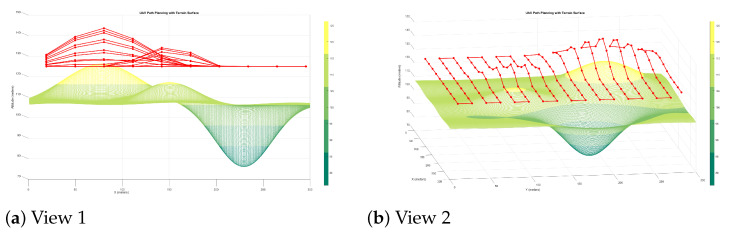
UAV coverage path using zoom-based CPP with reduced maximum focal length (0.01 m) over terrain with elevation changes; (**a**,**b**) present different viewpoints of the trajectory and coverage area.

To assess the feasibility of using a less capable or more cost-effective camera in the proposed system, a simulation was conducted with a significantly reduced maximum *f*. Specifically, the *f* range was modified to $f_{\min}=0.0043\;m$ and $f_{\max}=0.01\;m$, compared to the upper limit previously tested of 0.129 m. This cutback emulates the constraints of a lower-end PTZ camera. Figure 5 shows the UAV’s coverage path in terrain with different elevations, using a shorter focal range. Even though the zoom limits were stricter, the UAV was still able to keep the ground resolution the same by changing the *f* dynamically within the new limits. When it was not possible to obtain the desired resolution just by changing *f*, the system made up for it by changing the UAV’s altitude as needed.

**Figure 6:**
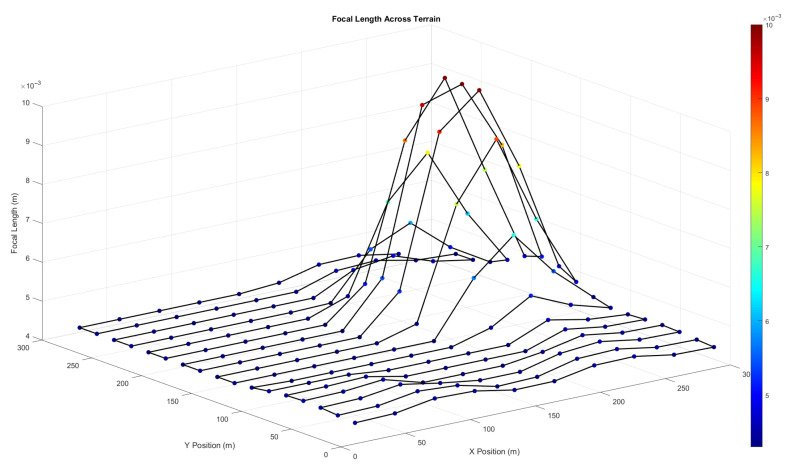
Focal length variation along the UAV flight path using a lower-end PTZ camera. The system compensates for limited zoom capability through altitude adjustments to maintain image quality.

Figure 6 shows the changes to *f* that happened along the path. As expected, the *f* reached the upper limit in places with a lot of elevation. This result indicates that a smaller zoom range increases the operational difficulty. Nevertheless, the system remained functional, and the image quality in the surveyed area was consistently high. This analysis validates the adaptability of the coverage planning algorithm to camera systems with narrower optical zoom capabilities. Although the reduced zoom range limits flexibility, without compromising mission objectives, demonstrating that the proposed model is robust enough to operate with more affordable camera hardware, provided that altitude adjustments are permitted.

### 3.5. Practical Considerations and Limitations

While the proposed zoom-aided coverage path planning (CPP) framework demonstrates strong performance in simulation, several practical considerations and limitations must be addressed for real-world deployment.

Impact of Rapid Zoom Changes: Frequent zoom adjustments, especially over steep or complex terrain, may introduce latency or image instability due to camera limitations or mechanical delays. In real-world scenarios, high zoom rates could lead to motion blur or reduced image sharpness if the gimbal stabilisation is inadequate or the PTZ system lacks fast autofocus capabilities. Therefore, smoothing or rate-limiting zoom transitions might be necessary in physical implementations.

UAV Hardware Compatibility: The method assumes real-time terrain data and direct control over PTZ camera zoom. This requires hardware compatibility with common UAV flight stacks such as ArduPilot or PX4, and camera modules that support low-latency zoom control through PWM or MAVLink. Systems with fixed focal lengths or limited zoom precision may not benefit fully from the proposed method. However, Section 3.4 demonstrates that even low-end systems can maintain coverage quality by combining limited zoom with altitude adjustments.

Environmental Conditions: The simulations presume ideal atmospheric conditions and precise terrain awareness. In practice, wind gusts, vibration, lighting variations, or GNSS signal loss can degrade imaging quality or compromise the UAV’s ability to follow planned paths. For example, a strong wind may cause drift or oscillations, which can affect the precision of zoom-based compensation. Sensor fusion techniques can mitigate these effects.

## 4. Conclusions

This work introduces a novel zoom-aided coverage path planning (CPP) simulation framework that leverages the dynamic focal length adjustment of UAV-mounted pan–tilt–zoom (PTZ) cameras to maintain consistent ground resolution across varying terrain elevations. Unlike traditional CPP strategies that rely solely on altitude modulation, the method implemented integrates real-time zoom control to achieve resolution uniformity while minimising energy consumption and flight complexity.

The proposed system performs effectively across a wide range of scenarios, as demonstrated through extensive simulations using synthetic terrains with different levels of difficulty. The zoom-based method covered the same area or more than just using altitude methods. It also made the flight path shorter by up to 26% in some cases and made the UAV’s path smoother. The system also worked well with lower-end cameras that did not zoom as much, demonstrating compatibility with a wide range of hardware setups.

In general, the zoom-aware CPP framework is a helpful and energy-efficient way to carry out UAV missions that require high-resolution pictures of uneven ground. It is suitable for both advanced and cost-effective PTZ systems, which makes it useful in various real-world situations, like precision farming, keeping an eye on the environment, and assessing disasters.

Future work will explore the integration of reinforcement learning (RL) to make the CPP framework more flexible and independent. RL is a promising way to help UAVs learn the best ways to cover an area in real time, especially when the area is unknown or changes quickly. By framing the path planning problem as a series of decisions, RL agents can learn to balance energy efficiency and resolution consistency by interacting with the environment directly.

In parallel, it is planned to validate the proposed CPP strategy in real-world conditions. Field trials with UAV-mounted PTZ cameras will be conducted to evaluate system performance under realistic environmental conditions, sensor uncertainties, and mechanical constraints. These experiments will support practical deployment and verify the robustness and energy-saving benefits demonstrated in simulation.

## Figures and Tables

**Table 1:** PTZ camera specifications used in Section 2.2.

Parameter	Value
Resolution	1920 × 1080 pixels
FOV (Horizontal)	63.7 deg
Zoom Capability	30× optical
Sensor Width	Assumed from resolution and FOV
Command response latency	<50 ms

**Table 2:** Performance comparison between altitude-based and zoom-based CPP for Terrain Type 1.

Implementation	Total Distance (m)	Total Flight Time (s)
Altitude-Based CPP [18]	4976.66	228.09
Zoom-Based CPP	4945.92	169.00

**Table 3:** Calculated minimum and maximum UAV-ground distances based on camera parameters.

Parameter	Value
Minimum UAV-ground distance	17.39 m
Maximum UAV-ground distance	521.80 m

## Data Availability

No new data were created or analyzed in this study. Data sharing is not applicable to this article.

## Abbreviations

The following abbreviations are used in this manuscript:

CPP	coverage path planning
*d*	UAV-to-ground vertical distance
*f*	focal length
$f_{\min}$	minimum focal length
$f_{\max}$	maximum focal length
FOV	field of view
GSD	ground sampling distance
$h_{\mathrm{base}}$	UAV base altitude
$h_{\mathrm{terrain}}$	terrain elevation
IBF	improved back and forth
PTZ	pan–tilt–zoom
$R_{v}$	camera vertical resolution in pixels
RL	reinforcement learning
ROI	region of interest
SAR	search and rescue missions
UAV	unmanned aerial vehicle
$w_{s}$	camera sensor width (m)
*Z*	UAV altitude
